# Effects on boar semen quality after infection with porcine reproductive and respiratory syndrome virus: a case report

**DOI:** 10.1186/1751-0147-55-16

**Published:** 2013-02-25

**Authors:** Martin Schulze, Sandra Revilla-Fernández, Friedrich Schmoll, Rudolf Grossfeld, Alfred Griessler

**Affiliations:** 1 IFN - Institute for Reproduction of Farm Animals Schönow e. V, 16321, Bernau, Germany; 2 AGES - Institute for Veterinary Disease Control Mödling, Austrian Agency for Health and Food Safety, 2340, Mödling, Austria; 3 Minitüb GmbH, 84184, Tiefenbach, Germany; 4 Traunkreis Vet Clinic, 4551, Ried im Traunkreis, Austria

**Keywords:** PRRSV, Boar, CASA, Semen quality, Spermatozoa

## Abstract

The effect of porcine reproductive and respiratory syndrome virus (PRRSV) on semen quality was examined in a group of 11 spontaneously infected boars in a commercial boar stud. Semen samples were collected 4 weeks prior to 4 weeks post-infection (wpi). Infection with PRRSV of the European genotype subtype 1 (EU-1) was verified by specific quantitative real-time polymerase chain reaction (RT-PCR) in 36% of the serum samples. All boars seroconverted before 4 wpi and remained in normal condition throughout the study. Comparison of the percentage of morphologically intact spermatozoa revealed an increase of acrosome-defective spermatozoa (*P* = 0.012) between −4 and 4 wpi. Significant deleterious effects on semen quality were detected for membrane integrity when semen had been stored for 2 days after sampling. Analysis of sperm subpopulations in a thermoresistance test on day 7 after sampling revealed alterations in the percentage of circular, progressively motile spermatozoa (*P* = 0.013), in the percentage of non-linear, progressively motile spermatozoa (*P* = 0.01), and on the amplitude of lateral sperm head displacement (*P* = 0.047). There was no difference in the incidence of mitochondrially active spermatozoa (*P* = 0.075). Investigation of routine production data between pre- and post-infection status showed no differences on ejaculate volume (*P* = 0.417), sperm concentration (*P* = 0.788), and percentage of motile spermatozoa (*P* = 0.321). This case report provides insights into a potential control strategy for PRRSV outbreaks in boar studs.

## Background

The porcine reproductive and respiratory syndrome virus (PRRSV) is one of the most important viruses in the swine industry worldwide [1]. PRRSV is a small enveloped RNA virus of the family *Arteriviridae*, of the order Nidovirales, and replicates in alveolar macrophages, monocytes, and macrophages of several other tissues including the male reproductive tract [2]. PRRSV was first described in North America in 1987 but isolated in 1990 in Europe [3]. Differences in virulence, and in antigenic and genetic properties led to the classification of PRRSV in European- (EU) and North American (NA) genotypes [4,5]. Both genotypes are able to induce chronic, persistent, and venereal infections [6].

High transmissibility is one of the key factors of PRRSV [7]. The virus can be transmitted in semen during the acute phase of infection and reaches the tissues of the reproductive tract by the dissemination of infected cells [8,9]. Furthermore, the duration of virus shedding in semen is quite variable in boars, especially between breeds, and ranges from days to months after infection. The shedding period may be independent of viremia or of the presence of neutralizing antibodies [10,11]. In semen, PRRSV has been detected as early as 2–3 days post infection (p.i.) [12] and up to 92 days p.i. [8].

PRRSV has been implicated in a broad range of clinical syndromes. Clinical signs in sows vary widely including respiratory distress, anorexia, abortion, premature farrowing, and a high number of mummified, stillborn, or weak piglets [13]. Differences in severity of the disease indicate that host factors are important for the persistence and pathogenesis of PRRSV [14]. Negative reproductive effects due to a PRRSV infection have aroused major attention to the possible role of boars in transmission as well as in the suspected viral effects on male fertility [15]. Literatures cite only a few studies about the effects of natural PRRSV infection on semen quality; meanwhile there are conflicting results regarding PRRSV effects on boars [3,16,17]. Commercial boar studs in Germany are therefore subjected to a surveillance program in order to recognize a possible PRRSV infection and to prevent transmission of the virus with distributed sperm doses.

In this case report, the effects of a spontaneous PRRSV infection on boar semen quality are described and a new component for a continuing PRRSV monitoring protocol in routine semen production is discussed.

## Case presentation

The boar stud, where the spontaneous PRRSV infection took place, was visited by independent auditors on a regular basis, following the guidelines of a quality management program. During these visits, all semen samples were collected before and after an outbreak of porcine reproductive and respiratory syndrome (PRRS). The exact time of the PRRSV infection remains unclear, and the antibody status of serum samples of the boars was determined retrospectively. The PRRSV infection in the boars was confirmed by ELISA, and semen evaluation results from infected boars were compared before and after infection. Present study was not an animal experiment. Therefore there is no requirement of disclosure or permit according to German law concerning animal shelter.

### Animals and semen processing

The artificial insemination (AI) center was a commercial production facility in Austria, holding 260 PRRSV seronegative boars at the start of the study period, including Pietrains, Landrace, Large White, and Duroc boars, which ejaculated weekly. All boars received commercial feed (pellets) for AI boars and were housed in individual pens equipped with nipple drinkers within the same building according to the European Commission Directive for Pig Welfare. Boars were dewormed twice a year and vaccinated against red murrain. The age (mean ± SD) of the boars was 24.5 ± 1.7 months (range: 10 to 36). The annual replacement rate was 50%. All boars were examined clinically daily.

As part of the routines at the AI center, weekly ejaculates were collected by gloved-hand method into plastic tubes, practice continued throughout the study period. The gel fraction of semen was removed by gauze filtration. Within 5 min after collection, semen volume and sperm concentration were determined to produce the insemination dose (90 ml in total), containing 2 × 10^9^ spermatozoa. Sperm concentration and motility were measured using the computer-assisted sperm analysis (CASA) system SpermVision™ (Minitube, Tiefenbach, Germany) with Leja-4 standardized counting chambers (Leja, Nieuw-Vennep, The Netherlands) according to the method described in the accompanying manual SpermVision™ (3.5). The semen was extended in Beltsville Thawing Solution (BTS, Minitube, Tiefenbach, Germany) at 32 ± 1°C and slowly cooled to 16°C over a 5 h period. After dilution and equilibration, two polyethylene insemination tubes (Minitube, Tiefenbach, Germany) per boar were transported to the Institute for Reproduction of Farm Animals Schönow (IFN, Bernau, Germany) while maintaining a temperature of 17 ± 2°C for further storage and analysis.

**Figure 1:**
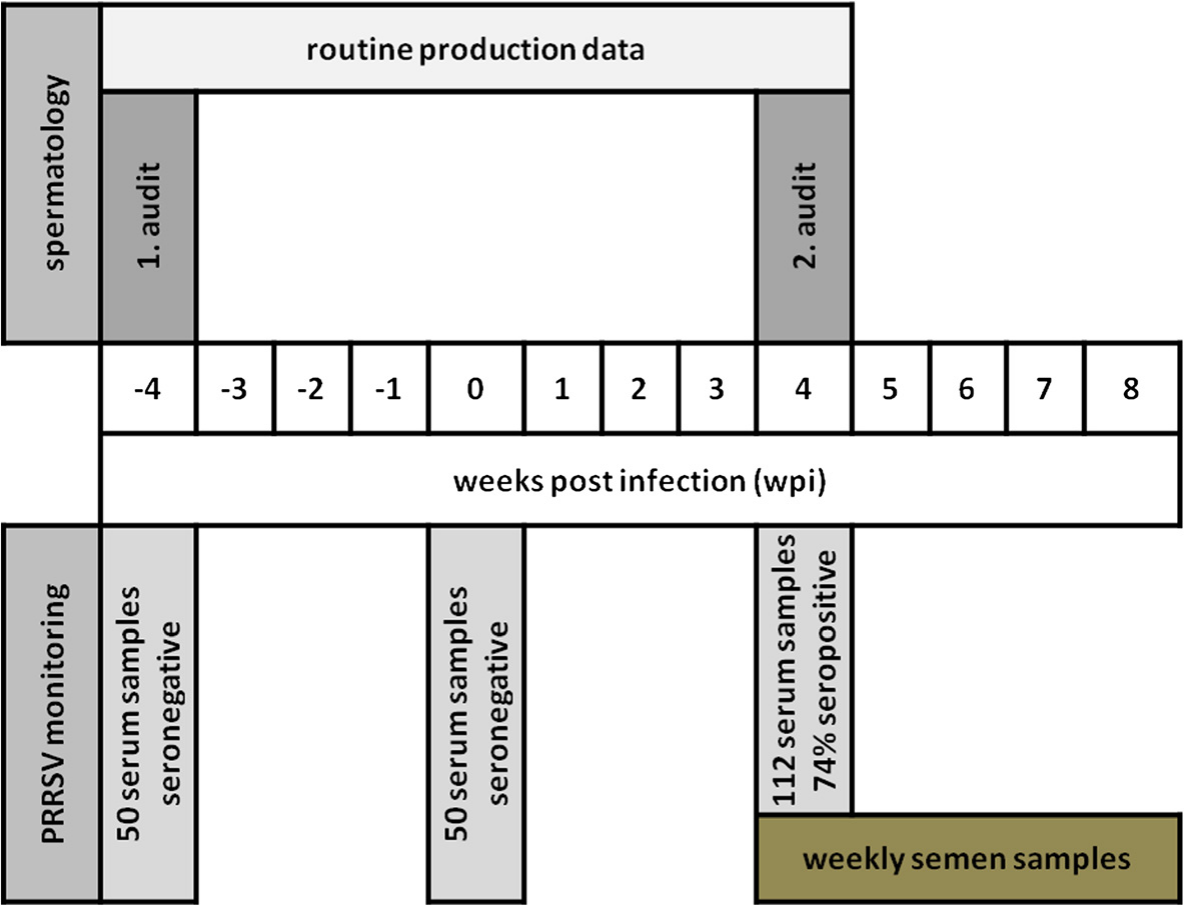
Time line of the field study.

Eleven PRRSV antibody negative Pietrain boars were randomly selected from the total population by a regular semen production audit of the IFN as a part of an external quality control program. Semen evaluation results from these boars were compared for the points in time of −4 and 4 wpi (Figure 1). Morphological analyses were performed on samples immediately after arrival at the IFN (d0). Flow cytometric mitochondrial activity and membrane integrity assessments were arranged on d2 of semen storage. On d7, a thermoresistance test (TRT) was performed.

### PRRSV antibody monitoring

Historically, the boar stud was seronegative for PRRSV. In early June 2012 though, the boar stud experienced an outbreak of PRRS. As part of the routine surveillance for PRRSV, 50 randomly selected serum samples had been collected monthly before the infection was observed, and additionally 112 serum samples were collected within the 4th wpi (Figure 1). The 11 boars of which semen samples were collected weekly were included in this group.

Nine milliliters of blood were drawn via jugular venipuncture using serum separation tubes. Samples were centrifuged at 950 g for 10 min. The serum was aliquoted into 2 ml plastic tubes and analyzed on the day of sampling. The IDEXX PRRS X3 Antibody Test Kit (IDEXX Switzerland AG, Liebefeld-Bern, Switzerland), a noncompetitive enzyme immunoassay, was used according to manufacturer instructions on detecting antibodies against EU and NA PRRSV strains.

### PRRS viral detection by RT-qPCR

As soon as the antibody test showed a positive result, quantitative real-time polymerase chain reaction (RT-qPCR) was performed to confirm the presence of the virus. For PRRS viral detection in semen by RT-qPCR, only the 11 boars of the semen production audit were investigated 4 wpi to 8 wpi (Figure 1).

RNA extraction from serum samples was performed by using the Freedom EVO® 150 (Tecan, Grödig, Austria) automated platform and the Nucleospin® 96 Virus kit (Macherey-Nagel, GenXpress, Wiener Neudorf, Austria) following manufacturer instructions.

RNA extraction from semen samples was performed immediately after arrival of the samples at the laboratory (approximately 2 hours after sampling) using an automated procedure and the Nucleospin® 96 RNA kit (Macherey-Nagel), by including only the cell fraction of the semen and by discharging the seminal plasma. Extraction efficiency and PCR inhibition were assessed by adding 5 μl of the internal control RNA to each sample, included in the commercial real-time PCR Kit prior to sample lysis.

Subsequently, all samples were analyzed with the TaqMan® PRRSV Reagents Kit (LifeTechnologies, Brunn am Gebirge, Austria) according to manufacturer instructions. This kit allows detection of and differentiation between NA and EU PRRSV genotypes and simultaneously amplification of the internal control. The thermo-profile, recommended by the kit protocol ABI 7500 Fast Real-Time PCR System (Life Technologies), was applied.

### Evaluation of sperm morphology

Semen samples were diluted 1:6 (vol/vol) in 1% PBS-buffered formalin to achieve 50 to 100 × 10^6^ sperms per ml. A 4-μl aliquot was transferred to a glass slide and covered by a slip. Sperm morphology was evaluated by counting 200 spermatozoa under phase-contrast at a magnification of 800 X (Jenaval, Carl Zeiss Jena, Jena, Germany). Morphological classification revealed the percentages of normal spermatozoa, abnormal heads, secondary apical ridge defects, deformed apical ridge, midpiece defects, proximal and distal cytoplasmic droplets, spermatozoa with a bent tail possessing a cytoplasmic droplet, as well as tail defects and multiple deformities.

### Evaluation of acrosome and membrane integrity

A triple-stain flow cytometric method consisting of propidium iodide (PI, Invitrogen™, Karlsruhe, Germany), FITC-labeled peanut agglutinin (FITC-PNA, Sigma, Deisenhofen, Germany), and FITC-labeled *Pisum sativum* agglutinin (FITC-PSA, Sigma, Deisenhofen, Germany) was applied to discriminate between viable and dead sperm cells, and to characterize membrane integrity in the acrosomal region: binding of FITC-PNA to the outer acrosomal membrane indicates a damaged cell membrane in the acrosome region; binding of FITC-PSA to the acrosomal content indicates damage to the acrosomal membrane. Stock solutions (1 mg/ml) of FITC-PNA and FITC-PSA were diluted 1:10 and 1:2 with PBS (26.8 mM Na_2_HPO_4_; 14.8 mM NaH_2_PO_4_; 102.6 mM NaCl; 305 ± 5 mOsmol/l; pH 7.0 at 20°C), respectively. Sperm samples were diluted (incubated) as follows: 375 μl BTS-preserved boar semen was mixed with 125 μl prewarmed 0.5% PBS-buffered formalin, with 12.5 μl FITC-PNA (incubation: 10 min), with 2.5 μl FITC-PSA (incubation: 10 min), and with 5 μl of 1.5 mM PI-solution (incubation: 5 min). After incubation for 10 min at 38°C in closed light proof tubes, aliquots of 10 μl stained-sperm suspension were resuspended in a 2 ml prewarmed PBS solution. The samples were measured in a flow cytometer (Partec GmbH, Münster, Germany) equipped with a 400 mW argon laser (Ex 488 nm), a 515–560 nm band-pass for FITC (green), and a 620 nm long-pass filter for PI (red). The system was triggered on the forward light scatter (FSC), and 15,000 cells per sample were characterized for their fluorescence at a flow rate of about 150 cells/s.

Subsequent to analyzing the sperm populations under scattered light and to transferring the outcomes to the two-parameter fluorescence diagram, four sperm subpopulations (Q1 to Q4) with different fluorescence characteristics were identified as follows: Q1: PI(+), PNA(−), PSA(−) = dead sperm with intact acrosomal region or complete loss of acrosomal region; Q2: PI(+), PNA(+), PSA(+) = dead sperm with defect acrosomal region; Q3: PI(−), PNA(−), PSA(−) = vital sperm with intact acrosomal region; and Q4: PI(−), PNA(+), PSA(+) = vital sperm with defect acrosomal region. Finally, percentages of proportions from Q3 were recorded.

### Evaluation of mitochondrial activity

Sperm viability and mitochondrial activity were assessed by the double-staining method rhodamine123/propidium iodide (R123/PI) using flow cytometry. A 250 μl aliquot of the diluted semen was mixed with 1 μl of a 0.13 mM R123-solution (R8004, Sigma-Aldrich, Taufkirchen, Germany), with 2.5 μl of a 1.5 mM PI-solution and was incubated under light exclusion for 20 min at 38°C (Techne Dri-Block® DB2.D, Techne AG, Burkhardtsdorf, Germany). After incubation, a 10 μl stained-sperm suspension was mixed with a 2 ml phosphate-buffered NaCl solution heated to 38°C. The samples were measured in the flow cytometer as described above. Results are presented as percentages of plasma membrane intact sperm cells with active mitochondria (PI negative and high fluorescence intensity for R123).

### Evaluation of sperm motility

Sperm motility of the BTS-preserved samples was assessed at the IFN according to the method described in the accompanying manual SpermVision™ (3.5) using the computer-assisted sperm analysis (CASA) system SpermVision™ (Minitüb, Tiefenbach, Germany), equipped with a phase contrast microscope (Olympus CX31), with a high-resolution chipcamera (60 frames/s) and with a heating platform (38°C). To assess sperm longevity at body temperature, a thermoresistance test (TRT) was performed [18,19]. Based on this procedure, an aliquot of 10 ml of each insemination dose was incubated at 38°C in a water bath (GFL 1002, Gesellschaft für Labortechnik, Burgwedel, Germany) for up to 300 min. Measurements were performed after 30 (TRT_1_) and 300 (TRT_2_) min of incubation respectively. In each case 2.4 μl of the samples were filled into a 38°C prewarmed “Leja-4“ chamber (Leja, Nieuw-Vennep, The Netherlands). Measurements occurred within approximately 15 to 30 s after filling the chamber. Analysis was based on the examination of 15 measurement fields per chamber with a total of 1,000 spermatozoa per sample.

The following parameters were recorded: progressively motile spermatozoa (%), linear, progressively motile spermatozoa (%), non-linear, progressively motile spermatozoa (%), circular, progressively motile spermatozoa (%), hyperactive, progressively motile spermatozoa (%), immotile spermatozoa (%), amplitude of lateral head displacement (ALH, μm), average orientation change of the head (AOC, °), beat cross frequency (Hz), distance curved line (μm), distance average path (DAP, μm), distance straight line (DSL, μm), velocity curved line (VCL, μm/s), velocity average path (VAP, μm/s), velocity straight line (VSL, μm/s), linearity (LIN, as a measure of a curvilinear path, VSL/VCL), straightness (STR, as the linearity of the average path, VSL/VAP), and wobble (oscillation measure of the actual path, VAP/VCL). Classification necessary for characterizing sperm motility was as follows: locally motile: DSL ≤ 4.5 μm; progressively motile: DSL > 4.5 μm; linearly motile: STR > 0.9, LIN > 0.5; non-linearly motile: STR < 0.9, LIN < 0.5; hyperactive: VCL > 80 μm/s, LIN < 0.65, ALH > 6.5 μm; circularly motile: DAP/radius ≥ 3, LIN < 0.5; immotile: AOC < 2.2°.

### Statistical analysis

For testing significant differences in weekly production data (volume, sperm concentration, and motility), non-parametric analysis according to Friedman was used. Semen evaluation results (morphology, acrosome/membrane integrity, mitochondrial activity, and thermoresistance test) were analyzed using the non-parametric Wilcoxon signed-rank test for assessing differences between pre- and post-infection status and applying the pre-infection values as control. Values are expressed as median and interquartile range (IQR). Results are represented as box-plot highest, respectively lowest values (Whisker), as inter-quartiles between quartiles 1 and 3 (box), and as median values. A two-tailed P-value of <0.05 was considered significant. Statistical analysis was carried out using the IBM SPSS Statistics 19 package (SPSS Inc., Chicago, IL).

### Clinical signs, PRRSV status, and findings by semen evaluation

All boars remained in normal condition throughout the study. All serum samples, including the samples of the 11 boars of the semen production audit, collected −4 to 0 wpi, tested negative for anti-PRRSV antibodies. Sera from 83 of 112 (74%) boars tested positive for anti-PRRSV antibodies 4 wpi, the 11 boars of which semen was examined in this case study included.

A PRRSV strain of the European genotype subtype 1 (EU-1) was detected by RT-PCR in 40 of 112 (36%) serum samples 4 wpi. RT-PCR quantitative values (Cq) ranged between 24.5 and 36.8. Of the 11 boars that seroconverted and whose semen was examined, four (36%) were found positive by RT-PCR 4 wpi. In the following weeks, analyses of semen samples of these boars generated punctual positive results for boar 2 (Cq 34.1 at 7 wpi) and for boar 10 (Cq 32.7 at 8 wpi).

**Figure 2:**
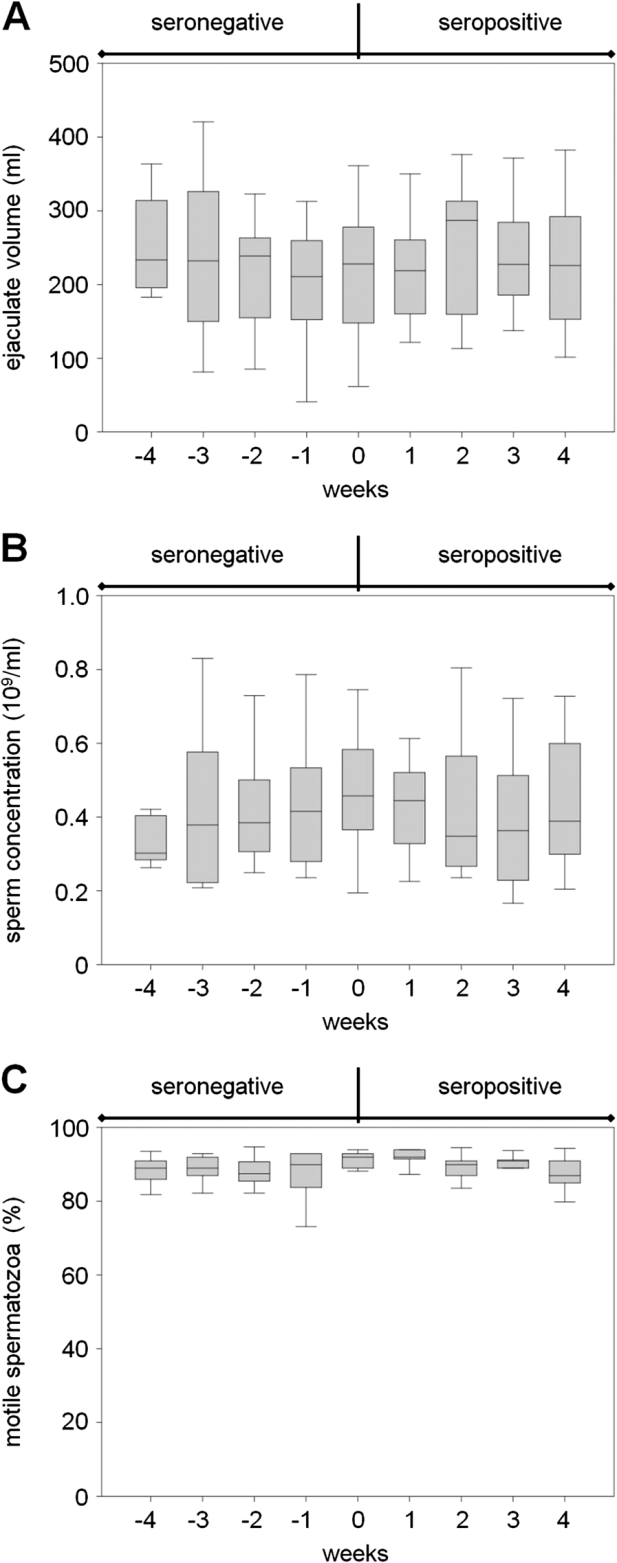
**Effect of a PRRSV infection in routine semen production.** Eleven boars were monitored over a period of −4 to 4 weeks post-infection (wpi) on the ejaculate parameter: volume (**A**), sperm concentration (**B**), and percentage of motile spermatozoa (**C**). Significant levels in ejaculate volume (*P* = 0.417), sperm concentration (*P* = 0.788), and motility (*P* = 0.321) between production weeks were not observed.

Analysis of routine production data, monitored over a period of −4 to 4 wpi, showed no significant differences between the weeks with respect to ejaculate volume (233 (176 to 280) ml, *P* = 0.417, Figure 2A), to sperm concentration (0.388 (0.283 to 0.507) × 10^9^ spermatozoa/ml, *P* = 0.788, Figure 2B), and to percentage of motile spermatozoa (90 (87 to 92) %, *P* = 0.321, Figure 2C). As expected, volume, sperm concentration, and motility varied widely over the study period. No effect on routine semen production was found after PRRSV exposure.

**Figure 3:**
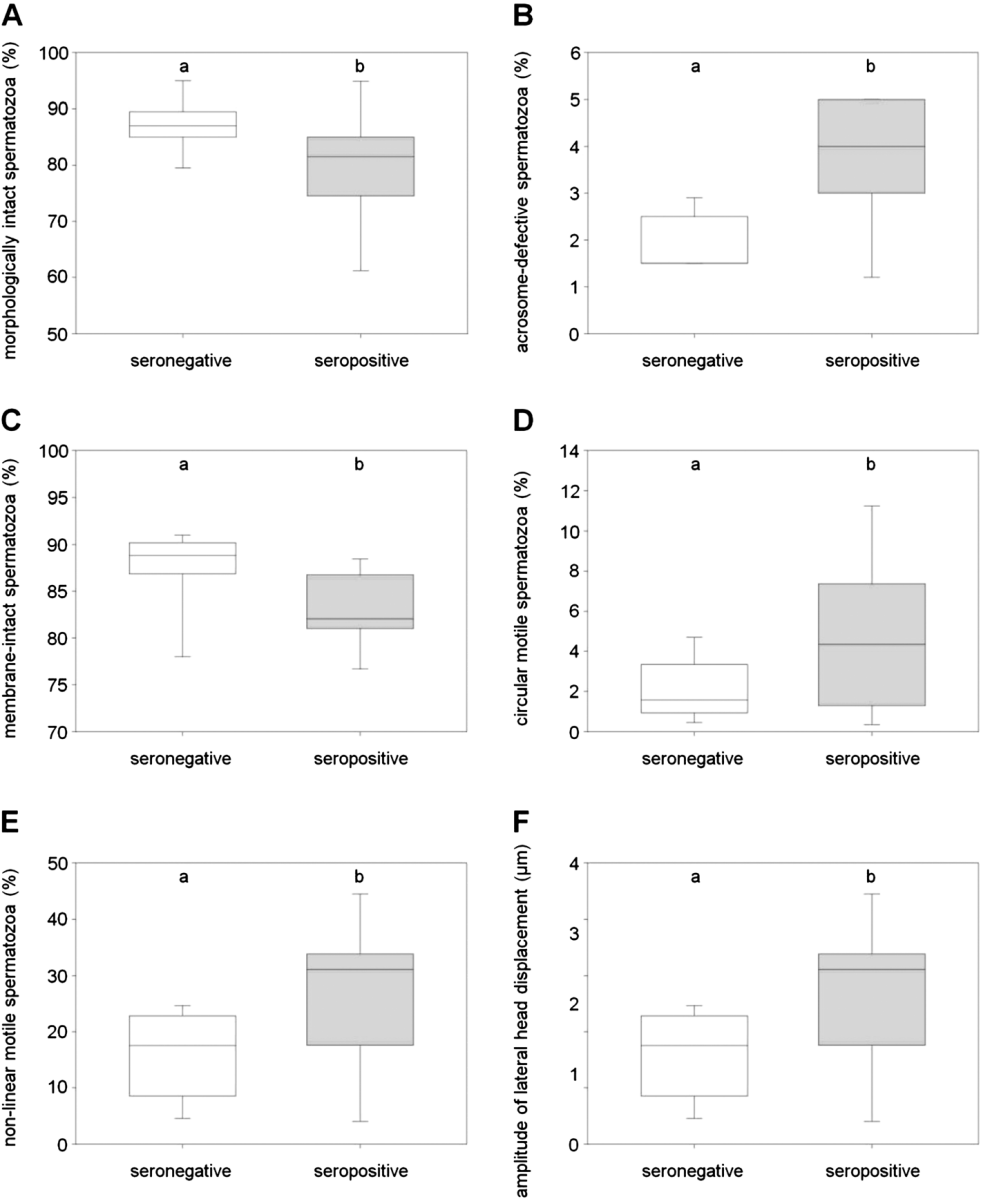
**Effect of a PRRSV infection on semen quality.** Eleven boars were examined −4 and 4 weeks post-infection (wpi) on the percentage of morphologically intact spermatozoa (**A**), on the percentage of acrosome-defective spermatozoa (**B**), on the percentage of membrane-intact spermatozoa (**C**), on the percentage of circular, progressively motile spermatozoa (**D**), on the percentage of non-linear, progressively motile spermatozoa (**E**), and on the amplitude of lateral sperm head displacement (**F**). The differences between −4 and 4 wpi were significant (**A**: *P* = 0.037; **B**: *P* = 0.012; **C**: *P* = 0.021; **D**: *P* = 0.013; **E**: *P* = 0.01; **F**: *P* = 0.047) and are referred to by different, lower-case letters (a, b).

Comparison of the percentages of morphologically intact spermatozoa on d0 showed significant differences (87.0 (85.5 to 88.8) % vs. 81.5 (77.0 to 81.9) %, *P* = 0.037, Figure 3A) between −4 and 4 wpi. A significant increase in acrosome-defective spermatozoa was observed (1.5 (1.5 to 2.3) % vs. 4.0 (3.0 to 4.8) %, *P* = 0.012, Figure 3B). Effects of PRRSV infection on semen quality were also detected regarding membrane status on d2 of semen storage. Sperm analysis showed a significant decrease in the percentage of membrane-intact spermatozoa after infection (88.8 (87.2 to 89.8) % vs. 82.0 (81.1 to 86.3) %, *P* = 0.021, Figure 3C).

During the TRT, no significant effect of PRRSV infection on the percentage of progressively motile spermatozoa was found. TRT was performed on d7 of semen storage for addressing sperm performance in the female genital tract after AI. No effect of the PRRSV infection of shorter (TRT_1_) or longer (TRT_2_) incubation at 38°C on sperm motility was found. Furthermore, there was no significant difference after PRRSV infection in the incidence of mitochondrially active spermatozoa (85.1 (81.7 to 86.2) % vs. 80.5 (79.6 to 81.8) %, *P* = 0.075) on d2 of semen storage. Separate analyses of the results from individual boars showed differences, some with alterations, while those from other boars remained within normal limits.

Analysis of sperm motion pattern in the TRT_2_ after 300 min of incubation at 38°C revealed significant alterations after PRRSV infection (Figure 3D-F). Percentage of circular, progressively motile spermatozoa (1.6 (1.1 to 3.0) % vs. 4.4 (2.2 to 6.6) %, *P* = 0.013, Figure 3D) increased significantly, together with the percentages of non-linear, progressively motile spermatozoa (17.5 (10.2 to 21.6) % vs. 31.1 (21.3 to 32.9) %, *P* = 0.01, Figure 3E). The non-linearity of progressive sperm motion reflected a significant influence of the PRRSV infection. Sperm in particular showed an increased non-linear movement as a function of the significant increase in the amplitude of lateral sperm head displacement (2.4 (2.1 to 2.5) μm vs. 2.8 (2.5 to 2.9) μm, *P* = 0.047, Figure 2F).

## Discussion

PRRSV infection in a commercial boar stud was detected during a routine quality audit and 11 randomly selected boars were examined in detail. Sera from these boars tested positive for the presence of PRRSV-specific antibodies at 4 wpi by ELISA and PRRSV subtype EU-1 was detected in 36% of the serum samples. The exact time of seroconversion remains unknown. However, PRRSV infection could be determined in two semen samples, at 7 and 8 wpi, respectively.

Clinical manifestation of an acute PRRS infection is usually mild or absent so that the presence of an acute infection may remain clinically unrecognized, as numerous literatures reflect [2,8,9,15]. However, anorexia, transient lethargy, depression, mild pyrexia, or loss of libido have been reported in some cases [16,17]. Clinical signs were not observed in the present study and all boars remained in normal condition throughout the study period. Lack of clinical signs is a risk factor for PRRSV transmission, as specific surveillance measurements are usually not used, i.e. routine analysis of serum samples by PCR during the peak of viremia or after seropositive results allowing detection of the shedding of PRRSV in semen.

Deleterious effects of PRRSV infection on ejaculate volume, sperm concentration, and motility were not observed in this study although having been reported previously [16]. Moreover, previous studies have demonstrated that experimental inoculation with a Spanish PRRSV EU-1 strain induced significant alterations in sperm quality, particularly in progressive motility and morphology, starting at 2 wpi. An increase in cytoplasmic droplets and a decrease in the percentage of spermatozoa with normal apical ridge have been recorded [20]. Adverse effects on morphology and alterations in progressive motility have also been described after experimental vaccination of boars by a modified-live virus vaccine [21]. Several other studies have shown that semen quality after infection with PRRSV was not affected [9,17].

The results of the current study show that spontaneous PRRSV infection had a significant effect on boar semen quality. For visualization of membrane defects, propidium iodide and fluorescent lectins FITC-PNA and FITC-PSA have been applied. Flow cytometry data revealed damage to membranes and acrosomes compared to pre-infection semen. The damaged sperm subpopulation consisted of a majority of PI permeable (dead) spermatozoa with concomitant membrane disturbance in the acrosomal region. Thus, differences in microscopically counted proportions of spermatozoa with acrosomal defects were verified. The number of spermatozoa with abnormal head, deformed apical ridge, midpiece defects, tail defects, and/or multiple deformities was not found significantly increased.

The results indicate that damaged spermatozoa occur due to a direct influence of the viral replication in the male reproductive tract, especially in spermatogonias, spermatids, and spermatocytes [22]. Infection of the spermatogenic epithelium is having a direct effect on spermatogenesis as well as causing cellular depopulation and desquamation. PRRSV has repeatedly been isolated from the head, body, and tail of the epididymis of experimentally infected boars [23]. Evidently, this tropism is very efficient for sexual transmission and contributes to reduced semen quality. In the present study, motility analyses demonstrated that the PRRSV infection had no significant influence on the percentage of progressively motile spermatozoa or on the incidence of mitochondrially active spermatozoa. Potential alterations in semen quality may be influenced by the viral genotype and strain.

An interesting finding of the present study was a significant change in sperm movements after PRRSV infection characterized by increasing ALH, coupled with an increased percentage of non-linear and circular progressively motile spermatozoa. Our observations suggest that alterations in sperm motion patterns and in membrane integrity are noticeable but, unfortunately, such alterations are non-specific diagnostic indicators of infection with PRRSV under field conditions. A threshold in semen motion pattern, i.e. non-linearity in sperm movement above a certain value, could help to detect abnormal semen motility and could subsequently stimulate an investigation into PRRSV infection. These control measures could include CASA systems that are used for quality assessment in routine semen production in an increasing number of boar studs. Thus, many additional semen quality parameters could be obtained during routine semen evaluation, but these data have not been efficiently exploited yet. A combination of CASA data with frequent PRRSV monitoring should be used in order to improve early detection of potential PRRS outbreaks.

## Conclusions

A spontaneous infection with PRRSV in boars induced a significant decrease of intact acrosomes and membranes in spermatozoa and, similarly, alterations in sperm motion patterns. PRRSV surveillance and monitoring in boar studs should not exclusively be based on clinical signs and serology. More extended control measures are essential for early detection of PRRSV or other infectious agents, especially when boars seroconvert from negative to positive serostatus.

## Competing interests

The authors declare that they have no competing interest.

## Authors’ contributions

MS performed the field work, collected the data, drafted the manuscript and contributed to the final manuscript preparation. SF and FS performed the virology and contributed to drafting the manuscript. RG contributed to drafting the manuscript. AG coordinated the work, sampled the boars and contributed to drafting the manuscript. All authors have read and approved of the final manuscript.
